# Ovarian clear cell carcinoma with or without endometriosis origin in a single institution cohort

**DOI:** 10.1007/s12672-023-00649-8

**Published:** 2023-04-01

**Authors:** Mingming Sun, Wei Jiang

**Affiliations:** 1 Department Gynecology, Obstetrics and Gynecology Hospital of Fudan University, 419 Fangxie Road, Shanghai, 200011 People’s Republic of China; 2 Shanghai Key Laboratory of Female Reproductive Endocrine Related Diseases, Shanghai, People’s Republic of China

**Keywords:** Ovarian clear cell carcinoma (OCCC), Endometriosis-origin, Clinical characteristics, Overall survival (OS), Progression-free survival (PFS), Lymphadenectomy

## Abstract

**Background:**

As ovarian clear cell carcinoma (OCCC) has distinct clinical features, biology, genetic characteristics and mechanisms of pathogenesis, and whether the origin of endometriosis or not affects the prognosis of OCCC remains controversial.

**Methods:**

We retrospectively collected medical records and follow-up data of patients with OCCC treated at the Obstetrics and Gynecology Hospital of Fudan University from January 2009 to December 2019. Further, we divided patients into 2 groups. Group 1: non-endometriosis origin; Group 2: endometriosis origin. Clinicopathological characteristics and survival outcomes were compared between the 2 groups.

**Results:**

A total of one hundred and twenty-five patients with ovarian clear cell carcinoma were identified and included. In the overall patients’ population, the 5 year overall survival was 84.8%, the mean overall survival was 85.9 months. The results of the stratified analysis showed that early stage (FIGO stage I/II) OCCC had a good prognosis. The results of univariate analyses indicated that a statistically significant relationship between overall survival (OS) and FIGO stage, lymph node metastasis, peritoneum metastasis, chemotherapy administration methods, Chinese herbal treatment, molecular target therapy. As for progression-free survival (PFS), a significant relationship between PFS and child-bearing history, largest residual tumor size, FIGO stage, tumor maximum diameter, lymph node metastasis was found, respectively. FIGO stage and lymph node metastasis are common poor prognostic factors affecting OS and PFS. The multivariate regression analysis revealed that FIGO stage (p = 0.028; HR, 1.944; 95% CI 1.073–3.52) and treatment by Chinese herbs (p = 0.018; HR, 0.141; 95% CI 0.028–0.716) were identified as influencing factors with regard to survival. The presence or absence of lymphadenectomy did not affect OS of 125 OCCC patients (p = 0.851; HR, 0.825; 95% CI 0.111–6.153).

There was a trend towards a better prognosis for patients with OCCC of endometriosis origin than those with OCCC of non-endometriosis origin (p = 0.062; HR, 0.432; 95% CI 0.179–1.045). The two groups differed with respect to several clinicopathological factors. And the proportion of patients with disease relapse was higher in Group 1 (46.9%) than in Group 2 (25.0%), with a statistically significant difference (p = 0.048).

**Conclusions:**

Surgical staging and treatment by Chinese herbs postoperatively are two independent prognostic factors affecting the OS of OCCC, early detection and Chinese herbal medicine combined with chemotherapy postoperatively may be a good choice. Tumor with endometriosis-origin was found less likely to relapse. While the non-necessity of lymphadenectomy in advanced ovarian cancer has been proven, the need for lymphadenectomy in the early stage ovarian cancer, including early stage OCCC, still deserved to be explored.

## Introduction

Epithelial ovarian cancers (EOCs) is one of the most common gynecologic malignancies with a high mortality rate. BRCA1/2 germline mutations are the strongest known genetic risk factors for EOCs and are found in 6–15% of women with EOC. The BRCA1/2 status can be used for patients’ counselling regarding expected survival, as BRCA1/2 carriers with EOC respond better than non-carriers to platinum-based chemotherapies. This yields greater survival, even though the disease is generally diagnosed at a later stage and higher grade [1]. EOCs are classified into type I and type II [2]; Of these, ovarian clear cell carcinoma (OCCC), endometrioid ovarian carcinoma, mucinous ovarian carcinoma and low-grade serous ovarian carcinoma are classified as type I, while type II is represented by high-grade serous ovarian cancer (HGSOC) [3]. The tumor that is currently classified as ovarian clear cell carcinoma was most likely originally described in 1899 by Peham as “hypernephroma of the ovary”, based on the striking similarity of the reported case to renal clear cell carcinoma [4]. Scully and Barlow’s seminal report [5] was also significant to detail a strong association between endometriosis and OCCC, and introduce the term clear cell carcinoma for these tumor. In 1973, ovarian clear cell carcinoma was included in the World Health Organization (WHO) classification of ovarian tumors [6].

Ovarian clear cell carcinoma (OCCC) is the second most common histological subtype, accounting for 5–25% of all EOCs [7, 8]. The prevalence of OCCC is largely region and ethnicity specific, it accounts for approximately 10% of EOCs in Europe and the United States with a higher incidence of about 10%-25% in Asian populations [9–11]. Compared to HGSOC, the most common type of EOC, OCCC has a younger onset, is more likely to be diagnosed in the early stage, is closely associated with endometriosis, and is characterized microscopically by a typical glycogen-filled clear cytoplasm and the presence of hobnail cells with a typical immunohistochemical phenotype [12–15]. OCCC has a unique genetic profile with a lower p53 mutation rate (25%) and a lower BRCA1/2 mutation rate (6.3%) but higher mutation rates in ARID1A, PIK3CA and PTEN compared to HGSOC [16–20]. Since inflammatory and epigenetic processes seem to play a predominant role in the pathogenesis of OCCC, immune checkpoint inhibitors and targeting the PI3K pathway as well as epigenetic treatment approaches may play an important role in the treatment of these tumor entities [21]. Current treatment recommendation for OCCC is based on data collected from cohort studies based on HGSOC, and surgery combined with postoperative platinum-based chemotherapy is the recommended option [22]. Moreover, we noticed that when mutations occur within DNA repair pathways, there is an increased risk of chemotherapy resistance. Given that a significant proportion of OCCC shows homologous recombination deficiency, they should be susceptible to PARP inhibitor therapy. Among PARP inhibitors, olaparib, rucaparib, and niraparib have been approved by the FDA and/or the EMA in EOC in different settings. Olaparib, rucaparib, and niraparib trap PARP approximately 100-fold more efficiently than veliparib [23]. Early stage OCCC has a better prognosis, while advanced/recurrent patients have a poor prognosis, which is related to their insensitivity to chemotherapy and chemoresistance [24, 25].

More and more studies have confirmed that OCCC and ovarian endometrioid carcinoma are all have close relationships with ovarian endometriotic cysts, which originate from atypical endometrial cells and or possibly endometriotic cells [26–28]. Common mutations in OCCC are frequently found in benign endometriosis without malignant lesions, including ARID1A, PIK3CA, PPP2R1A and KRAS. In particular, deletion of ARID1A gene (BAF250a) frequently occurs in atypical endometriosis, which indicates an early role in carcinogenesis [17, 18, 29]. It indicated that endometriosis as the tissue origin of OCCC, they have shared genomic abnormalities and monoclonal relationships (most likely atypical ovarian endometriotic cysts), that OCCC may be caused by malignant transformation of endometriosis with a common genetic pedigree, that known oncogenes cause malignant transformation of ovarian endometriotic epithelium, and the microenvironment of endometriosis also promotes carcinogenesis [30, 31]. In addition, it has been suggested that overexpression of HNF-1β was detected in OCCC and 40% of benign endometriotic cysts [28, 32, 33]. And biological properties such as PD-L1 overexpression and copy number variation (CNV) may promote the cancerous transformation in ovarian endometriosis from a non-invasive precursor lesion to OCCC [34–36]. As OCCC has distinct clinical features, biology, genetic characteristics and mechanisms of pathogenesis, as well as the dilemma of insensitivity to chemotherapy, and the exact pathogenesis of ovarian endometriosis to OCCC has not been fully elucidated, further research and exploration are still needed. In this study, we retrospectively collected medical records and follow-up data of patients with OCCC from a single center, particularly those with OCCC of endometriosis and non-endometriosis origin, try to trigger more thinking about the future management of OCCC.

## Materials and methods

### Patients

Between January 2009 and December 2019, 139 patients were diagnosed with ovarian clear cell carcinoma and treated at Obstetrics and Gynecological Hospital of Fudan University, China. This study was approved by the ethics committee of OB/GYN Hospital of Fudan University. Data were collected from electronic medical records and outpatient visits. All eligible patients had a pathological diagnosis of ovarian clear cell carcinoma in various stages, women with a concurrent malignancy were excluded. In all, 125 women were enrolled in this study.

### Data collection

Data collected included demographic information, clinical, surgical and pathological information, chemotherapy information and follow-up information. Following a electronic medical records search, baseline data were obtained from the database of patients’ medical records and included age at diagnosis, BMI, menopause, parity, personal medical history, comorbid medical disease; Clinical data were also obtained from the patients and included symptom, preoperative tumor markers level, presence or absence of endometriosis, manifestations of endometriosis, duration of endometriosis, whether there is ascites, imaging findings; Surgical and pathological details included surgery mode, complete or incomplete surgery (complete surgical procedure consisted of total hysterectomy, bilateral salpingo-oophorectomy, omentectomy, pelvic and para-aortic lymphadenectomy, and debulking procedures such as colon resection), fertility-sparing surgery (the preservation of the uterus and one adnexa), largest residual tumor size, surgical staging (the International Federation of Obstetrics and Gynecology, FIGO staging system), tumor maximum diameter, peritoneal cytology, lymph node metastasis (preoperative evaluation of retroperitoneal swollen lymph nodes was confirmed by computed tomography and MRI and/or PET-CT), omentum metastasis, peritoneum metastasis, tumor origin (histologically confirmed), postoperative pathological and immunohistochemical results (The pathologic diagnosis was performed and supervised independently by 2 pathologists). Adjuvant therapy (observation or adjuvant chemotherapy, treatment by Chinese herbs, molecular target therapy), chemotherapy circles (adjuvant chemotherapy regimen was a platinum based doublet: carboplatin (AUC = 5–6) and paclitaxel (135–175 mg/m^2^) every 3 weeks, for 3–6 cycles). Platinum-sensitivity was defined as relapse occurring ≥ 6 months after the completion of last regimen or lack of recurrence and platinum-resistance was defined as relapse occurring within 6 months of the completion of last regimen. Patients came back to our hospital for follow-up evaluation with the interval of 3 months for the first 2 years, with the interval of 6 months for the next 3 years, and annually thereafter. We also collected date of disease progression or death, disease progression details, adjuvant therapy after disease progression, status of the patient at the most recent follow-up. Overall survival (OS) and progression-free survival (PFS) was calculated from the date of primary surgery to death and disease progression/recurrence, respectively, or the last disease-free visit.

### Analysis

The survival analysis was based on the Kaplan–Meier method, and the results were compared using the log-rank test. Cox regression analysis was used to determine factors affecting survival and recurrence, and results are presented as HRs with 95% CIs. The distributions of clinicopathological factors were evaluated using the Student’s t-test or the χ^2^-test as appropriate. Spearman’s correlation analysis was used to assess the correlation between endometriosis origin and clinicopathological characteristics of OCCC patients. Multivariate survival analysis was performed using Cox regression model including prognostic factors that were significant in univariate analysis. all p values reported are two-tailed and a p < 0.05 was considered significant. All statistical analyses were performed using Statistical Program for Social Sciences (SPSS) (version 19.0).

## Result

In all, 125 women were surgically diagnosed with OCCC at Obstetrics and Gynecology Hospital of Fudan University during the study period. The characteristics of patients with OCCC involved are shown in Tables 1, 2. Besides, information on the clinical characteristics of the relapsed patients among all OCCC patients is presented in Table 3.

**Table 1:** Patients' characteristics in 125 ovarian clear cell carcinoma women and Univariate analyses of impact of various prognostic parameters on overall survival (OS) (p1) and recurrence (Progression-free survival, PFS) (p2)

Characteristics	Number of cases (%)	Univariate analysis					
Age, median (range), year	50 (29–79)	*p1*	Hazard Ratio	95% confidence interval	*p2*	Hazard Ratio	95% confidence interval
≤ 60 y, n (%)	98 (78.4)	0.586	1.323	0.484–3.618	0.425	0.706	0.300–1.661
> 60 y, n(%)	27 (21.6)						
BMI (Kg/m^2^)							
≤ 24.0	85 (68.0)	0.781	0.874	0.339–2.254	0.761	0.885	0.404–1.939
> 24.0	40 (32.0)						
Menopause							
Yes	67 (53.6)	0.521	1.328	0.559–3.156	0.998	1.001	0.493–2.032
No	58 (46.4)						
Parity							
Parous	109 (87.2)	0.224	0.508	0.170–1.513	0.024	0.313	0.114–0.855
Nulliparous	16 (12.8)						
Ovarian cancer family history							
Yes	2 (1.6)	0.763	0.049	0.000–16,550,195.08	/	/	/
No	123 (98.4)						
Breast cancer history							
Yes	4 (3.2)	0.531	1.904	0.253–14.317	0.563	0.553	0.074–4.116
No	121 (96.8)						
Symptom							
Vaginal bleeding	6 (4.8)	0.834	1.027	0.801–1.317	0.436	0.913	0.726–1.148
Menstrual change	3 (2.4)						
Abdominal pain/bloating	19 (15.2)						
Pelvic mass	71 (56.8)						
Combination	1 (0.8)						
None	1 (0.8)						
Others	24 (19.2)						
Comorbid medical disease							
Yes	50 (40.0)	0.334	0.627	0.243–1.616	0.32	1.496	0.676–3.311
No	75 (60.0)						
Endomotriosis disease history							
Yes	52 (41.6)	0.457	0.708	0.285–1.758	0.511	1.274	0.619–2.622
No	73 (58.4)						
Manifestations (Types) of endometriosis							
Ovarian endometriotic cyst	57 (45.6)	0.132	1.268	0.931–1.727	0.888	0.983	0.775–1.247
Peritoneal endometriosis	4 (3.2)						
Deep infiltrating endometriosis (DIE)	0						
None	64 (51.2)						
Pretreatment CA-125 (U/mL)							
< 35	38 (30.4)	0.114	2.405	0.809–7.152	0.301	1.61	0.654–3.966
≥ 35	83 (66.4)						
Unknown	4 (3.2)						
Pretreatment neutrophil percentage							
Rise	40 (32.0)	0.621	0.827	0.390–1.755	0.434	0.784	0.426–1.443
Normal	78 (62.4)						
Decrease	7 (5.6)						
Pretreatment lymphocyte percentage							
Rise	2 (1.6)	0.908	1.052	0.446–2.480	0.916	0.959	0.440–2.091
Normal	83 (66.4)						
Decrease	40 (32.0)						
Imaging findings							
Positive	124 (99.2)	0.722	20.589	0.000–3.519E8	/	/	/
Negative	1 (0.8)

**Table 2:** Surgical and pathological characteristics in 125 ovarian clear cell carcinoma women and univariate analyses of impact of various prognostic parameters on overall survival (OS) (p1) and recurrence (Progression-free Survival, PFS) (p2)

Characteristics	Number of cases (%)	Univariate analysis					
Referred after incomplete surgery		p1	Hazard Ratio	95% confidence interval	p2	Hazard Ratio	95% confidence interval
Yes	23 (18.4)	0.167	0.243	0.033–1.808	0.48	1.69	0.394–7.255
No	102 (81.6)						
Complete staging surgery							
Yes	111 (88.8)	0.426	2.26	0.303–16.850	0.967	1.044	0.140–7.798
No	14 (11.2)						
Lymphadenectomy							
Yes	118 (94.4)	0.851	0.825	0.111–6.153	/	/	/
No	6 (4.8)						
Unknown	1 (0.8)						
Surgery mode							
Laparoscopy	74 (59.2)	0.854	1.085	0.455–2.584	0.461	0.762	0.370–1.570
Laparotomy	50 (40.0)						
Unknown	1 (0.8)						
Fertility-sparing surgery							
Yes	2 (1.6)	0.715	0.048	0–549,846.541	/	/	/
No	123 (98.4)						
Largest residual tumor size							
Residual mass ≤ 1.0 cm	123 (98.4)	0.715	20.745	0–2.366E8	0.014	32	2.002–511.602
Residual mass > 1.0 cm	1 (0.8)						
Unknown	1 (0.8)						
FIGO stage							
I	89 (71.2)	0.001	2.013	1.334–3.038	0.001	2.186	1.382–3.459
IA	19 (15.2)						
IB	1 (0.8)						
IC	69 (55.2)						
	53 (42.4)						
	13 (10.4)						
	3 (2.4)						
II	11 (8.8)						
III	21 (16.8)						
IV	2 (1.6)						
Unknown	2 (1.6)						
Tumor maximum diameter (mm)							
≤ 50	16 (12.8)	0.122	1.682	0.870–3.251	0.029	0.559	0.332–0.943
50–100	46 (36.8)						
> 100	60 (48.0)						
Unknown	3 (2.4)						
Ascites							
Presense	46 (36.8)	0.25	1.618	0.712–3.674	0.698	0.874	0.443–1.724
Absence	74 (59.2)						
Unknown	5 (4.0)						
Peritoneal cytology							
Positive	25 (20.0)	0.058	1.725	0.982–3.031	0.449	0.844	0.545–1.308
Negative	44 (35.2)						
Unknown/unexamined	56 (44.8)						
Lymph node metastasis							
Yes	12 (9.6)	0.002	5.454	1.830–16.251	0.01	3.312	1.336–8.210
No	109 (87.2)						
Unknown	4 (3.2)						
Omentum metastasis							
Yes	12 (9.6)	0.907	1.111	0.189–6.536	0.145	3.436	0.653–18.084
No	111 (88.8)						
Unknown	2 (1.6)						
Peritoneum metastasis							
Yes	20 (16.0)	0.01	3.453	1.343–8.877	0.214	1.641	0.751–3.586
No	103 (82.4)						
Unknown	2 (1.6)						
Tumor origin							
Endometriosis	70 (56.0)	0.062	0.432	0.179–1.045	0.341	1.413	0.694–2.880
Non-endometriosis origin	55 (44.0)						
Progression time (endometriosis to OCCC), year							
≤ 5	21 (30.0)	0.127	0.194	0.026–1.474	0.258	2.511	0.827–7.624
5–10	16 (22.9)						
> 10	12 (17.1)						
Unknown	21 (30.0)						
Neoadjuvant chemotherapy							
Yes	9 (7.2)	0.943	0.929	0.124–6.950	0.646	1.333	0.391–4.549
No	116 (92.8)						
Intraoperative chemotherapy use							
Yes	98 (78.4)	0.193	0.379	0.088–1.632	0.53	0.713	0.248–2.047
No	25 (20.0)						
Unknown	2 (1.6)						
Adjuvant chemotherapy							
Yes	119 (95.2)	0.551	21.376	0.001–496,996.629	/	/	/
No	6 (4.8)						
Adjuvant chemotherapy cycles							
< 6 courses	47 (37.6)	0.986	1.006	0.501–2.022	0.097	2.015	0.882–4.606
≥ 6 courses	73 (58.4)						
Unknown	5 (4.0)						
Chemotherapy administration methods							
Intravenous	60 (48.0)	0.044	2.381	1.025–5.533	0.193	0.624	0.307–1.268
Intravenous + Intraperitoneal	55 (44.0)						
Unknown	10 (8.0)						
Chemotherapy-related side-effects							
Yes	97 (77.6)	0.173	1.4	0.863–2.274	0.777	0.929	0.558–1.546
No	4 (3.2)						
Unknown	24 (19.2)						
Treatment by Chinese herbs							
Yes	38 (30.4)	0.012	0.154	0.036–0.663	0.538	1.29	0.574–2.899
No	66 (52.8)						
Unknown	21 (16.8)						
Molecular target therapy*							
Yes	11 (8.8)	0.001	4.819	1.862–12.477	0.161	0.57	0.259–1.252
No	114 (91.2)

**Table 3:** Relapsed patients' characteristics and Univariate analyses of impact of various relapse-related prognostic parameters on overall survival

Characteristics	Number of cases (%)	Univariate analysis		
Disease relapse		p	Hazard Ratio	95% confidence interval
Yes	38 (30.4)	/	/	/
No	71 (56.8)			
Unknown	16 (12.8)			
Progression/ Relapse time*, months				
≤ 6	11 (28.9)	0.829	1.047	0.691–1.586
> 6	22 (57.90)			
Unknown	5 (13.2)			
Tumor Origin				
Non-endometriosis Origin	23(60.5)	0.581	0.785	0.333–1.852
Endometriosis Origin	15(39.5)			
Progression/ Relapse manifestations				
Elevated tumor markers	10 (26.3)	0.71	1.074	0.738–1.561
Local mass based on imaging	9 (23.7)			
Metastasis based on imaging or pathology	12 (31.6)			
Others	1 (2.6)			
Unknown	6 (15.8)			
Chemo-resistance				
Yes	12 (31.6)	0.18	0.636	0.328–1.233
No	22 (57.9)			
Unknown	4 (10.5)			
Treatment after progression/ relapse**				
Chemotherapy	23 (60.5)	0.256	0.811	0.566–1.164
Surgery	6 (15.8)			
Molecular target therapy	1 (2.6)			
Treatment by Chinese herbs	1 (2.6)			
Alleviative/palliative treatment	3 (7.9)			
Unknown	4 (10.5)			
Molecular target therapy				
Yes	11 (28.9)	0.082	2.22	0.903–5.456
No	27 (71.1)			

Table 1 shows us the clinical baseline information of the OCCC patients. The mean age at diagnosis was 50 years (range, 29–79 years). 68% of them had a BMI of less than or equal to 24.0 kg/m^2^. About half of the patients are menopausal (53.6%). 87.2% patients had history of delivery. There were only 2 (1.6%) patients had ovarian cancer family history and only 4 (3.2%) patients had breast cancer history. The most common clinical symptom of OCCC in our study was pelvic mass (56.8%). Pelvic masses are adnexal masses of undetermined origin, benign or malignant, found by the patient or by clinical examination or by imaging tests such as ultrasound, CT, MRI, PET/CT. Of these patients, 52 (41.6%) had a previous history of endometriosis disease. Followed by the most common ovarian endometriotic cyst, 4 patients had peritoneal endometriosis and no one had deep infiltrating endometriosis in the series. Preoperative CA-125 values elevated (≥ 35U/ml) in 83 (66.4%) cases. And among them, 38 cases had normal levels of CA-125. Neutrophil and lymphocyte percentages in pretreatment blood tests were in the normal range in most patients, but the neutrophil percentage tended to rise and the lymphocyte percentage tended to decline. Positive imaging findings account for almost all cases (99.2%). Further, a detailed description of the surgical and pathological characteristics is shown in Table 2. 102 (81.6%) patients with OCCC undergone the primary surgery at our institution and only 23 of them referred after incomplete surgery. Most cases (88.8%) had gone through complete surgical staging procedures and only 2 of them had fertility sparing surgery in hopes of preserving fertility, other patients underwent conservative surgery due to severe complications that excluded them from complete surgical staging. Lymphadenectomy was omitted or replaced by lymph node biopsy in 6 cases because of advanced stage or patient morbidity, and in addition, those who only undergone lymph node biopsy or para-aortic lymphadenectomy without undergoing pelvic lymphadenectomy were not included in the lymphadenectomy group, but a negative biopsy was considered as no lymph node metastasis, a positive para-aortic lymph node was regarded as positive lymph node metastasis. Seventy-four women were treated by laparoscopy, fifty had laparotomy. Upon the surgery, ascites were present in 46 cases and peritoneal cytology was positive in 25 (20.0%) cases while 56 (44.8%) records were unavailable. Tumor diameter with ≤ 50 mm took up 12.8%, 50–100 mm in 46 (36.8%) and > 100 mm in 60 (48.0%) cases. Debulking surgery with residual tumor ≤ 1.0 cm (R0) was achieved in 98.4% of cases. During the procedure, 78.4% of patients received intraoperative chemotherapy, mainly cisplatin. Early-stage disease predominated, the surgical stage was I/II in 100 (80.0%) and III/IV in 23 (18.4%) patients. Among stage I patients, stage IC accounted for the majority (69/89). After reviewing the pathological records of these patients, a total of 70 (56.0%) tumors arose from endometriosis based on the criteria of Sampson and Scott [37] [the criteria include: (1) the coexistence of benign and malignant tissue in the same ovary which have the same histologic relationship to each other as in endometrial carcinoma of the uterine corpus; (2) the carcinoma must actually be seen to arise in this tissue, and not to be invading it from some other source; (3) and additional supportive evidence includes the finding of tissue resembling endometrial stroma surrounding characteristic epithelial glands, and the finding of old hemorrhage rather than fresh, since the latter can be the result of trauma resulting from surgical manipulation. (4) a microscopic section must show the benign endometriosis running into and continuous with the malignant epithelium]. 12 (9.6%) patients had positive lymph node metastases. Twelve of 125 women had omentum metastasis. Peritoneum metastasis occurred in 20 (16.0%) patients. Endometriosis progressed to OCCC within 5 years in 21 patients, representing 30% of the total number. Only 9 patients received neoadjuvant chemotherapy as assessed by their general condition and preoperative Suidan’s CT score [38]. After surgery, 119 (95.2%) patients received a first-line combined chemotherapy with a platinum-based regimen. 58.4% of patients received at least 6 courses of chemotherapy, while 47 (37.6%) patients received lesser courses because of intolerance of side effects or uncomplaisance. Intravenous chemotherapy alone or intravenous combined with intraperitoneal chemotherapy each accounts for approximately half of the postoperative chemotherapy population. The main chemotherapy-related side-effects were manifested as different degrees of myelosuppression (77.6% of the patients). Through our follow-up, we found that 30.4% of the patients received post-operative herbal treatments to regulate their bodies and achieved certain results. Of all patients, only 11 (8.8%) patients received molecular target therapy (mainly with bevacizumab treatment), and they were mainly patients with advanced and relapsed disease. Only one patient received immunotherapy by joining the clinical trial, but stopped the therapy due to the serious side effects and is still alive. None underwent postoperative radiotherapy.

As is shown in Table 3, of the women with follow-up, 38 (30.4%) OCCC patients presented with disease relapse. And among them, 11 (28.9%) had refractory disease, 12 (31.6%) had chemo-resistant disease, and 22 (57.9%) met the criteria for chemo-sensitive disease. 60.5% of recurrent patients were of non-endometriosis origin. The most common manifestation of recurrent disease was imaging-indicated metastases lesions or pathological evidence of metastases (31.6%), followed by elevated tumor markers (26.3%) and local mass based on imaging (23.7%). These patients were also followed for treatment after relapse, chemotherapy remained the mainstay of treatment after relapse (60.5%). A significant number of women (15.8%) had undergone surgical procedure again, primarily to relieve tumor load and remove isolated lesions. It was noted that among the relapsed patients, 11 of them received chemotherapy and molecular target therapy (mainly with bevacizumab treatment) at the same time.

**Fig. 1:**
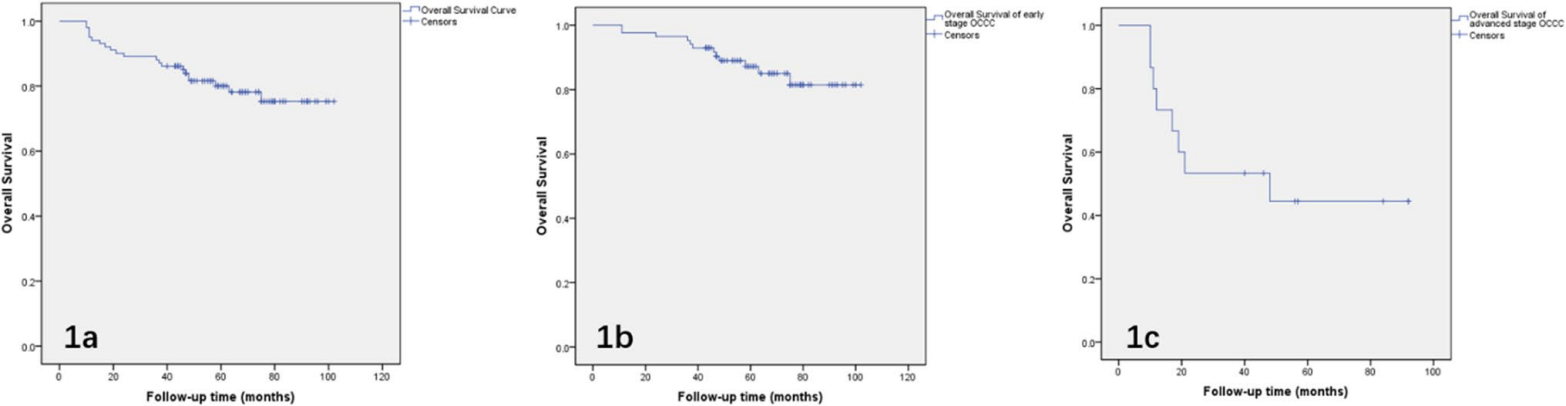
**a. **Survival curves of overall survival; **b**. Survival curves of overall survival in early stage OCCC (FIGO stage I and II); **c**. Survival curves of overall survival in advanced stage OCCC (FIGO stage III and IV)

**Fig. 2:**
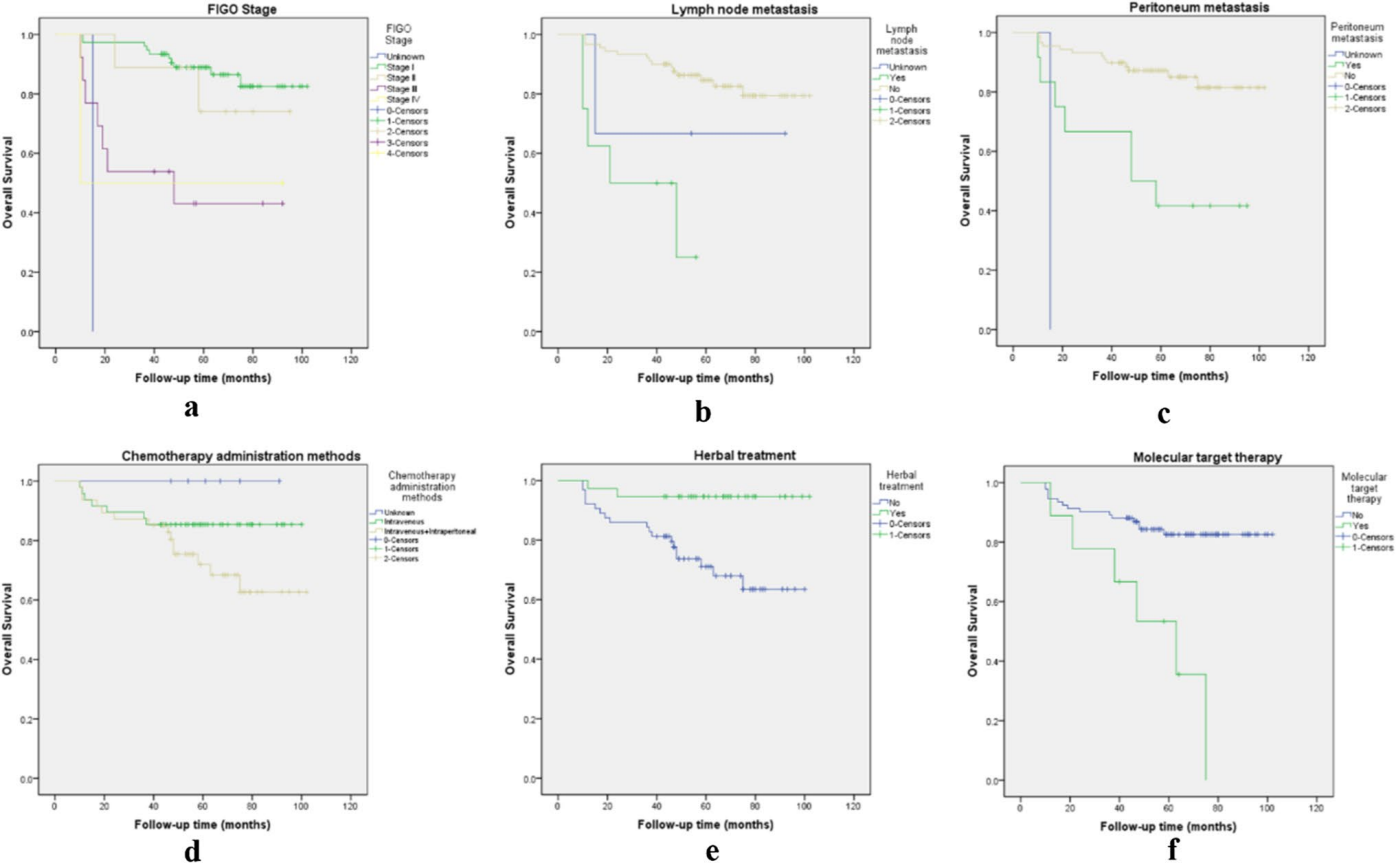
(**a**, **b**, **c**, **d**, **e**, **f**). Survival curves of prognostic factors for overall survival by FIGO stage (**a**), lymph node metastasis (**b**), peritoneum metastasis (**c**), chemotherapy administration methods (**d**), Chinese herbal treatment (**e**), molecular target therapy (**f**)

**Fig. 3:**
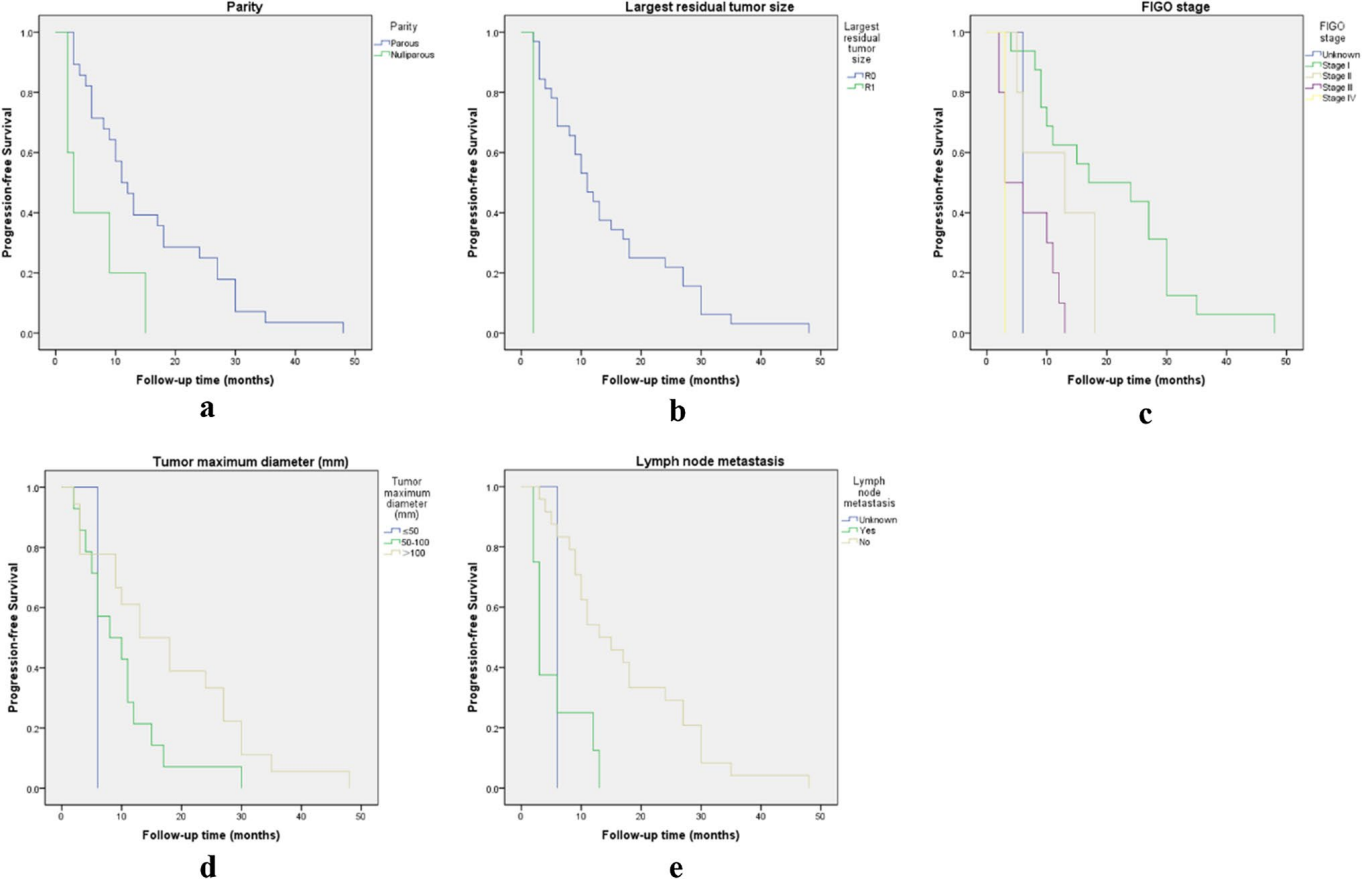
(**a**, **b**, **c**, **d**, **e**). Survival curves of prognostic factors for progression-free survival by child-bearing history (**a**), largest residual tumor size (**b**), FIGO stage (**c**), tumor maximum diameter (**d**), lymph node metastasis (**e**)

Survival analysis was retrospectively performed to identify the significant outcome predictors that affect disease relapse and survival in patients with OCCC. In the overall patients’ population, the 5 year overall survival was 84.8%, the mean overall survival was 85.9 months (95% CI 79.7–92.1). The median follow-up time from the initial surgery was 58.0 months (range, 10–102 months) (Fig. 1a**)**. We also performed survival analysis for early (stage I-II) and advanced (stage III-IV) stage OCCC respectively, and the results are shown in Fig. 1b and Fig. 1c. Early stage OCCC had a good prognosis, the mean overall survival was 91.9 months (95% CI 86.5–97.2). In comparison, the mean overall survival of advanced OCCC was 51.8 months (95% CI 32.7–71.0), the median overall survival for advanced OCCC was 48 months. A detailed description of the results of univariate analyses on overall survival (p1) and progression-free survival (p2) is shown in Tables 1, 2, it indicated that a statistically significant relationship between survival probability and FIGO stage (p = 0.001), lymph node metastasis (p = 0.002), peritoneum metastasis (p = 0.01), chemotherapy administration methods (p = 0.044), Chinese herbal treatment (p = 0.012), molecular target therapy (p = 0.001), the survival curves of these factors affecting OS are shown in Fig. 2(a, b, c, d, e, f). Among the many characteristics, peritoneal cytology, tumor origin are two clinical factors, which had p values less than 0.1 for univariate analysis of OS, then, we also included these two clinical data in the subsequent multivariate analyses. For analysis of the correlation between clinical data and PFS, a significant relationship between PFS and child-bearing history (p = 0.024), largest residual tumor size (p = 0.014), FIGO stage (p = 0.001), tumor maximum diameter (p = 0.029), lymph node metastasis (p = 0.01) was found, respectively (Fig. 3a, b, c, d, e). The results of multivariate analyses carried out to determine the effect of demographic characteristics and clinical features on overall survival are provided in Table 4

**Table 4:** Multivariate analyses of significant prognostic parameters on overall survival in patients with ovarian clear cell carcinoma cox-regression analysis.

Characteristics	Wald	p	Harzard ratio	95% confidence interval
FIGO stage	4.809	0.028	1.944	1.073–3.52
Peritoneal cytology	1.609	0.205	0.651	0.336–1.263
Lymph node metastasis	0.167	0.683	0.762	0.207–2.81
Peritoneum metastasis	2.154	0.142	2.414	0.744–7.834
Tumor origin	1.26	0.262	0.54	0.184–1.584
Chemotherapy administration methods	3.555	0.059	2.402	0.966–5.974
Treatment by Chinese herbs	5.587	0.018	0.141	0.028–0.716
Molecular target therapy	6.275	0.012	4.009	1.353–11.880

Through our analyses, it revealed that FIGO stage and lymph node metastasis are common poor prognostic factors affecting OS and PFS. Overall survival decreased in patients who developed peritoneum metastases (p = 0.01; HR, 3.453; 95% CI 1.343–8.877), but there was no significant difference in the effect on PFS. Patients treated with intravenous combined with intraperitoneal chemotherapy have a worse prognosis than those treated with intravenous chemotherapy alone (p = 0.044; HR, 2.381; 95% CI 1.025–5.533). Interestingly, in terms of treatment, in addition to post-operative chemotherapy, patients treated with herbal remedies have a better OS (p = 0.012; HR, 0.154; 95% CI 0.036–0.663). However, patients receiving bevacizumab-based molecular target therapy have a poorer prognosis (p = 0.001; HR, 4.819; 95% CI 1.862–12.477). In our analysis, women who have given birth to offspring have a lower risk of disease recurrence (p = 0.024; HR, 0.313; 95% CI 0.114–0.855). Larger tumor diameter was associated with prolonged PFS (p = 0.029; HR, 0.559; 95% CI 0.332–0.943). Whether surgery achieved R0 was associated with recurrence and did not affect OS. A subsequent multivariate regression analysis revealed that FIGO stage (p = 0.028; HR, 1.944; 95% CI 1.073–3.52) and treatment by Chinese herbs (p = 0.018; HR, 0.141; 95% CI 0.028–0.716) were identified as risk factors with regard to survival. Patients who received molecular target therapy were mainly patients with advanced and relapsed disease as mentioned above, so we ignored this factor even though the p value is less than 0.05.

**Fig. 4:**
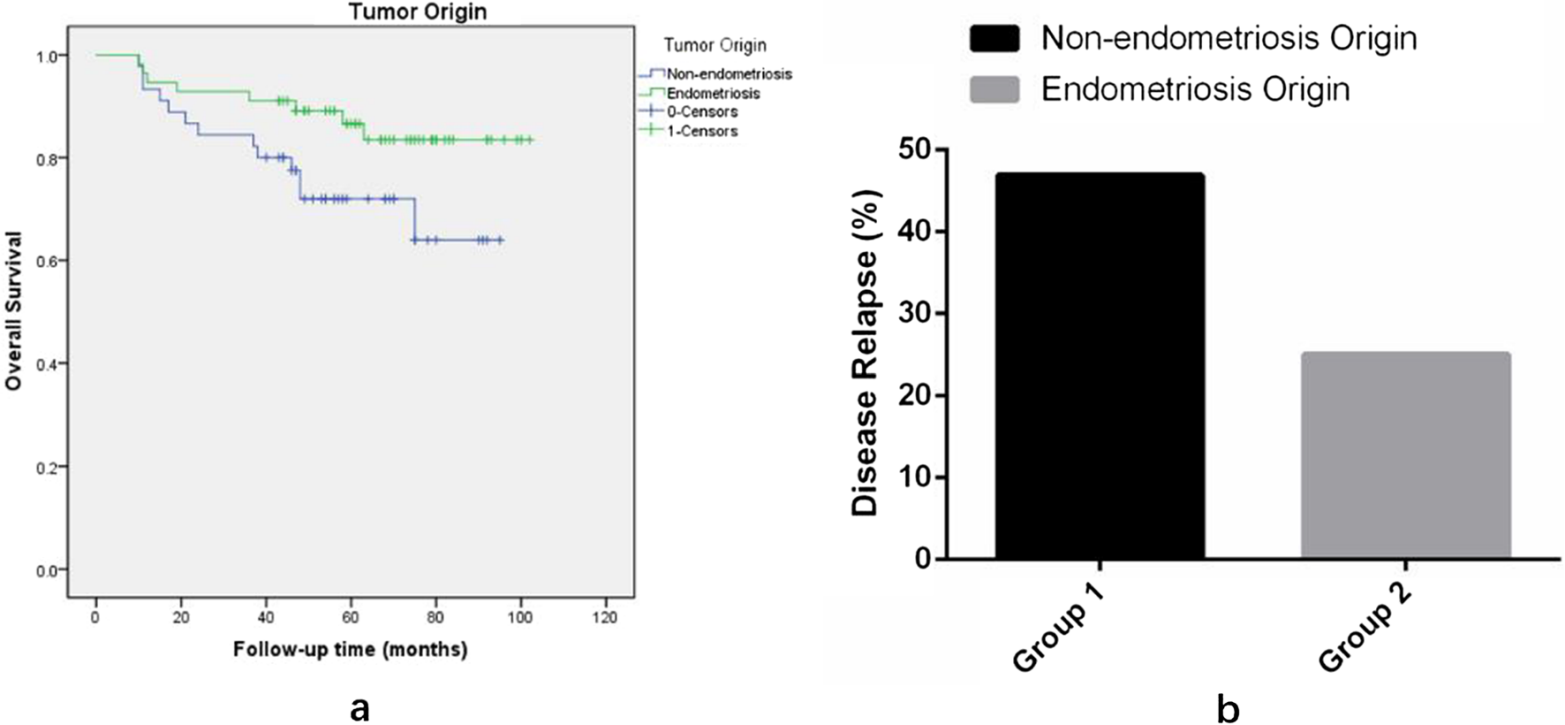
**a** Survival curves of prognostic factors for overall survival by Tumor origin; **b**. The proportion of patients with disease relapse in Group 1 (non-endometriosis origin) and Group 2 (endometriosis origin)

Besides, as is shown in Fig. 4a, we can see that even there was no significant difference between tumor origin and OS, a trend towards a better prognosis for patients with OCCC of endometriosis origin than those with OCCC of non-endometriosis origin (p = 0.062; HR, 0.432; 95% CI 0.179–1.045). To further evaluate the significance of endometriosis origin on the recurrence and prognosis of ovarian clear cell carcinoma (OCCC) and its relationship with other clinical parameters, we divided patients into 2 groups according to the association between ovarian endometriosis and OCCC on pathology. The patients were classified as Group 1 (non-endometriosis origin) if the tumor was not originated from endometriosis. The patients were classified as Group 2 (endometriosis origin) if clear cell carcinoma arose from ovarian endometriosis or if ovarian endometriosis was present and found elsewhere in the ovary. Clinicopathological characteristics and survival outcomes were compared between the 2 groups. The two groups differed with respect to clinicopathological factors, such as age, menopause status, endometriosis disease history, manifestations of endometriosis, pretreatment CA-125 level, referred after incomplete surgery, peritoneal cytology and disease relapse. Of 125 OCCC patients at OB/GYN Hospital of Fudan University during the study period, 70 (56%) patients had OCCC arising from ovarian endometriosis or coexisting ovarian endometriosis elsewhere in the ovary, and 55 (44%) of these patients had OCCC of non-endometriosis origin. The patients’ baseline characteristics and clinico-surgical pathological characteristics between the two groups are presented in Table 5. Group 1 patients were older than Group 2 (p < 0.001), and most OCCC in postmenopausal patients did not have endometriosis origin (Group1), while those with endometriosis origin (Group 2) often appear before menopause (p < 0.001). Having analyzed our data, we have concluded that the majority of patients in Group 2 (70%, p < 0.001) have a history of endometriosis and their presentation mainly appeared as ovarian endometriotic cysts (75.7%, p < 0.001). There were more abnormal CA-125 levels in Group 1 patients than in Group 2 patients prior to surgery (80% vs 55.7%). No differences were found between the two groups in the number of patients underwent complete staging surgery. However, more patients in Group 2 referred after incomplete surgery (p = 0.004). Optimal debulking surgery, which was defined as the size of the largest residual tumor less than or equal to 1.0 cm, was performed in both groups, with 98.2% of patients in Group 1 and 98.6% of patients in Group 2 (p = 0.357). A higher percentage of patients in Group 1 had positive ascites cytology compared to Group 2 (42.1% vs 29.0%). More patients with OCCC of endometriosis origin (Group 2) were in the early stage of cancer (stage I and II, 87.1% vs 73.6%) than patients with OCCC of non-endometriosis origin, advanced-stage diseases (stage III and IV) were more frequent in Group 1 (26.4% vs 12.9%), but among stage I patients, stage IC patients accounted for a greater proportion of Group 2 patients (84.2% vs 65.6%). As for the data on other clinical-surgical pathological features, no differences were observed between the two groups in BMI, parity, symptom, pretreatment neutrophil and lymphocyte percentage, surgery mode, tumor maximum diameter, ascites presence, lymph node metastasis, omentum metastasis, peritoneal metastasis. After surgery, 53 patients (96.4%) in Group 1 and 66 patients (94.3%) in Group 2 received adjuvant chemotherapy (p = 0.59). No differences in chemotherapy cycles, chemotherapy administration methods, chemotherapy-related side-effects, Chinese herbs’ treatment and molecular target therapy were observed between the two group. It is worth noting that the proportion of patients with disease relapse was higher in Group 1 (46.9%) than in Group 2 (25.0%), with a statistically significant difference (p = 0.048), and as is depicted in Fig. 4b, this result is consistent with the previously mentioned the trend of higher 5-year OS in endometriosis origin OCCC patients (Group 2) compared with non-endometriosis origin patients (Group 1) (Fig. 4a), even though there was no statistically significant difference between the two groups in terms of PFS (p = 0.341). And, there were no significant differences in progression/ relapse time, progression/relapse manifestations, chemo-resistance and treatment after progression/relapse demonstrates Table 6 the results of spearman correlation analysis between endometriosis origin of OCCC and clinical indicators of each parameter.

**Table 5:** Comparison of 125 OCCC with and without endometriosis origin

Characteristics	Tumor Origin		p-value
	Group 1 (non-endometriosis origin)	Group 2 (endometriosis origin)	
Age, median (range), y	57 (29–79)	47 (29–67)	
≤ 60 y, n (%)	33	65	< 0.001
> 60 y, n(%)	22	5	
BMI (Kg/m^2^)			
≤ 24.0	37	48	0.877
> 24.0	18	22	
Menopause			
Yes	44	23	< 0.001
No	11	47	
Parity			
Parous	50	59	0.271
Nulliparous	5	11	
Tubal ligation history			
Yes	2	0	0.108
No	53	70	
Ovarian cancer family history			
Yes	0	2	0.206
No	55	68	
Breast cancer history			
Yes	2	2	0.806
No	53	68	
Symptom			
Vaginal bleeding	2	4	0.133
Menstrual change	3	0	
Abdominal pain/bloating	10	9	
Pelvic mass	26	45	
Combination	0	1	
None	0	1	
Others	14	10	
Endomotriosis disease history			
Yes	3	49	< 0.001
No	52	21	
Manifestations (Types) of endometriosis			
Ovarian endometriotic cyst	4	53	< 0.001
Peritoneal endometriosis	2	2	
Deep infiltrating endometriosis (DIE)	0	0	
None	49	15	
Pretreatment CA-125 (U/mL)			
< 35	11	27	0.009
≥ 35	44	39	
Unknown	0	4	
Pretreatment neutrophil percentage			
Rise	17	23	0.232
Normal	37	41	
Decrease	1	6	
Pretreatment lymphocyte percentage			
Rise	0	2	0.424
Normal	38	45	
Decrease	17	23	
Imaging findings			
Positive	55	69	0.373
Negative	0	1	
Referred after incomplete surgery			
Yes	4	19	0.004
No	51	51	
Complete staging surgery			
Yes	51	60	0.217
No	4	10	
Pelvic lymphadenectomy			
Yes	50	68	0.266
No	4	2	
Unknown	1	0	
Surgery mode			
Laparoscopy	29	45	0.259
Laparotomy	25	25	
Unknown	1	0	
Largest residual tumor size			
Residual mass ≤ 1.0 cm	54	69	0.357
Residual mass > 1.0 cm	0	1	
Unknown	1	0	
FIGO stage			
I	32	57	0.053
IA	10	9	0.297
IB	1	0	
IC	21	48	
II	7	4	
III	13	8	
IV	1	1	
Unknown	2	0	
Tumor maximum diameter (mm)			
≤ 50	4	12	0.058
50–100	16	30	
> 100	33	27	
Unknown	2	1	
Ascites			
Presense	26	20	0.098
Absence	27	47	
Unknown	2	3	
Peritoneal cytology			
Positive	16	9	0.011
Negative	22	22	
Unknown/unexamined	17	39	
Lymph node metastasis			
Yes	8	4	0.236
No	45	64	
Unknown	2	2	
Omentum metastasis			
Yes	8	4	0.061
No	45	66	
Unknown	2	0	
Peritoneum metastasis			
Yes	11	9	0.542
No	43	60	
Unknown	1	1	
Progression time (endometriosis to OCCC), year			
≤ 5	1	21	< 0.001
> 5	0	28	
Unknown	54	21	
Neoadjuvant chemotherapy			
Yes	5	4	0.468
No	50	66	
Intraoperative chemotherapy use			
Yes	45	53	0.125
No	8	17	
Unknown	2	0	
Adjuvant chemotherapy			
Yes	53	66	0.59
No	2	4	
Adjuvant chemotherapy cycles			
< 6 courses	20	27	0.756
≥ 6 courses	32	41	
Unknown	3	2	
Chemotherapy administration methods			
Intravenous	29	31	0.644
Intravenous + Intraperitoneal	22	33	
Unknown	4	6	
Chemotherapy-related side-effects			
Yes	40	57	0.101
No	4	0	
Unknown	11	13	
Treatment by Chinese herbs			
Yes	14	24	0.512
No	32	34	
Unknown	9	12	
Molecular target therapy*			
Yes	6	5	0.724
No	38	52	
Unknown	11	13	
Disease relapse			
Yes	23	15	0.048
No	26	45	
Unknown	6	10	
Progression/ Relapse time*, months			
≤ 6	6	5	0.881
> 6	14	8	
Unknown	3	2	
Progression/ Relapse manifestations			
Elevated tumor markers	3	7	0.202
Local mass based on imaging	7	2	
Metastasis based on imaging or pathology	8	4	
Others	1	0	
Unknown	4	2	
Chemo-resistance			
Yes	7	5	0.821
No	13	9	
Unknown	3	1	
Treatment after progression/relapse			
Chemotherapy	13	10	0.726
Surgery	4	2	
Molecular target therapy	1	0	
Treatment by Chinese herbs	0	1	
Alleviative/palliative treatment	2	1	
Unknown	3	1

**Table 6:** Spearman analysis of correlation between endimetriosis origin and clinicopathological characteristics of OCCC patients

Variables	With or without endometriosis origin	p-value
	Spearman correlation	
Age	− 0.497	< 0.001
BMI	0.005	0.956
Menopause	0.469	< 0.001
Parity	0.098	0.275
Tubal ligation history	0.144	0.109
Ovarian cancer family history	− 0.113	0.209
Breast cancer history	0.022	0.808
Symptom	− 0.088	0.332
Endomotriosis disease history	− 0.65	< 0.001
Manifestations (Types) of endometriosis	− 0.687	< 0.001
Pretreatment CA-125	0.224	0.013
Pretreatment neutrophil percentage	0.028	0.758
Pretreatment lymphocyte percentage	< 0.001	0.998
Imaging findings	0.08	0.378
Referred after incomplete surgery	0.255	0.004
Complete staging surgery	0.11	0.22
Pelvic lymphadenectomy	− 0.105	0.245
Surgery mode	− 0.107	0.237
Largest residual tumor size	0.079	0.382
FIGO stage	− 0.227	0.012
Tumor maximum diameter (mm)	− 0.237	0.009
Ascites	0.183	0.041
Peritoneal cytology	0.267	0.003
Lymph node metastasis	0.153	0.094
Omentum metastasis	0.157	0.084
Peritoneum metastasis	0.099	0.278
Progression time (endometriosis to OCCC), y	0.228	0.111
Neoadjuvant chemotherapy	0.065	0.472
Intraoperative chemotherapy use	0.113	0.213
Adjuvant chemotherapy	− 0.048	0.593
Adjuvant chemotherapy cycles	− 0.036	0.693
Chemotherapy administration methods	0.084	0.373
Chemotherapy-related side-effects	− 0.235	0.018
Treatment by Chinese herbs	0.113	0.254
Molecular target therapy*	− 0.077	0.442
Disease relapse	− 0.229	0.017
Progression/relapse time*, months	− 0.179	0.318
Progression/relapse manifestations	− 0.354	0.043
Chemo-resistance	− 0.017	0.921
Treatment after Progression/relapse	− 0.08	0.647
Survival State	0.2	0.039

## Discussion

Many factors can influence and indicate the prognosis of OCCC. With the development of technologies of proteomics, such as mass spectrometry (MS) and protein array analysis, the available novel biomarkers, namely, targeted proteomics, is a key technique that enables the validation and verification of biomarkers that have been discovered. It works with untargeted proteomics to complete the cycle of biomarker discovery and validation. Peptidomics, is the second new sub-division of proteomics and can, also, be used to shed light on new biomarkers. Further, exosomes, play a critical role in intercellular communication and they have emerged as a compelling diagnostic and prognostic biomarkers for OCCC, as they may transport some tumour-associated proteins [39]. And many studies have shown that the clinicopathological stage of the tumor is the most important prognostic factor for OCCC [10, 40]. Other poor prognostic factors include lymphatic vascular invasion, blocked p16 expression, deletion of BAF250a expression, β-catenin nuclear expression, abnormal p53 staining patterns, expression of IMP3, CBX7, Emi1, CXCR4, HOXA10, Glypican 3, MET gene amplification, CCNE1 copy number gain, MDM2 amplification in TP53 wild type cases and multiple somatic copy number variants [41–45].Our studies have suggested important roles of surgical staging and treatment by Chinese herbs postoperatively as two independent prognostic factors. Efficacy and safety of Chinese herbal medicine on ovarian cancer after surgery have been discussed in these years [46]. Researchers found that Chinese herbal medicine treatments significantly improved symptoms and enhanced curative effects. It also showed the unique superior chemotherapy tolerance in quality of patient’s life and minimal toxic and adverse effects due to chemotherapy [47]. Specifically, Chinese herbal medicine combined with chemotherapy after surgery may reduce incidences of gastrointestinal reactions, marrow depression, urinary system symptoms and regulate even boost the immune system [48, 49]. Therefore, when we are keep thinking ovarian cancer for improving outcomes, we should consider proper treatments that are truly palliative and improve symptom control [50]. And treatment should be stratified in accordance not only to prognosis, but also with more emphasis being placed on patients’ experience and on minimizing side effects, for all these reasons, Chinese herbal medicine combined with chemotherapy postoperatively may be a good choice.

What deserved our attention is that among the early stage (FIGO stage I/II) OCCC patients in our study, 97% (97/100) of them underwent lymphadenectomy and 1 patient had lymph node biopsy. And after the confirmation of the final pathology report, we found that of the 23 recurrent patients in early stage, 22 patients who underwent lymphadenectomy did not develop lymph node metastasis. Moreover, the presence or absence of lymphadenectomy did not affect OS of 125 OCCC patients (p = 0.851; HR, 0.825; 95% CI 0.111–6.153) by our data analysis. A prospective randomized controlled study of the effect of lymphadenectomy on survival in early-stage ovarian cancer found that although more positive lymph nodes were detected with systematic lymphadenectomy than with lymph node sampling, the study was not statistically valid enough to analyze the effect of systematic lymphadenectomy on PFS and OS in early-stage ovarian cancer due to the small sample size, and perioperative morbidity and postoperative complications were significantly higher in the systematic lymphadenectomy group than in the lymph node sampling group [51]. According to the previous literature [52], the complication rate of retroperitoneal systematic lymphadenectomy ranges from 5.9% to 24%, with the more serious recent complications occurring in 5.9% to 18.1% of these. Common recent complications include vascular injury, lymphatic cysts, small bowel obstruction and deep vein thrombosis, and possibly urinary fistula and postoperative infection, etc. The main long-term complication is lymphoedema of the lower limbs, which sometimes seriously affects the patient’s quality of life. In addition, lymph nodes are peripheral immune organs located on the way of lymphatic vessels, and their main function is to filter lymphatic fluid and produce immune cells, which participate in the immune response of the body. Although removal of regional lymph nodes blocks one of the metastatic pathways of tumors, it also weakens the anti-tumor immunity of the body. Besides, the functional protection of the immune organs contributes to the immunotherapy of tumors. The role of regional lymph nodes in the tumor immune cycle is crucial, as it is the site of initiation and maintenance of the body’s anti-tumor immune response, and its lack of function will cause a disconnect in the tumor immune cycle, bringing about a failure of immune supervision [53]. Whether systematic lymphadenectomy provides a survival benefit for patients with early-stage ovarian cancer, including those with early-stage OCCC, remains controversial. As mentioned previously in this study, patients with early stage OCCC have a good prognosis which is consistent with previous studies [7, 11, 13]. All these suggest that we may be able to omit lymphadenectomy in early stage OCCC patients, thereby reducing the risk of intraoperative injury, shortening the operative time and reducing the risk of postoperative complications associated with lymphadenectomy itself, and ultimately improving the patients’ postoperative quality of life to some extent. As the results of the various retrospective studies were inconsistent [54–57] and retrospective studies are vulnerable to the effects of bias from confounding factors, in China, there is an ongoing prospective multicenter randomized controlled study on “the Exemption of early-stage epithelial ovarian cancer from systemic lymphadenectomy”, and our institution, as one of the subcenters, is actively enrolling suitable patients for this clinical study. This multicenter clinical study aims to optimize treatment strategies in the future for early-stage ovarian cancer (including early-stage OCCC) and to provide a new evidence-based basis for updating clinical guidelines. In our study, all 12 patients with positive lymph nodes were advanced stage (FIGO stage III/IV) patients. The rate of positive lymph node metastases is approximately 52.2% (12/23). Even though patients with lymph node metastases had shorter OS (Fig. 2b) and PFS (Fig. 3e) among the 125 OCCC patients. After our stratified analysis of advanced stage OCCC, we found that lymph node metastasis had no significant effect on OS and PFS in patients with advanced disease, and the difference was not statistically significant (OS: p = 0.311; HR, 1.922; 95% CI 0.544–6.792; PFS: p = 0.937; HR, 1.058; 95% CI 0.261–4.287). We also analyzed whether lymphadenectomy affected OS in patients with advanced disease as well, and found that lymph node dissection did not affect OS in these advanced OCCC patients (p = 0.636; HR, 1.666; 95% CI 0.201–13.808). Here, our findings are consistent with those of the LION study [58] recently published in the New England Journal. The LION study suggested that lymphadenectomy did not result in longer PFS or OS in patients with advanced ovarian cancer when there were no clinically suspicious abnormal lymph nodes. According to LION, systemic lymphadenectomy does not provide a survival benefit for patients with advanced ovarian cancer whose lymph nodes are visual normal, but increases the risks and complications of surgery; Systematic lymphadenectomy should not be routinely performed in these patients and international guidelines have been rewritten as a result [22].

Previous studies have reported conflicting outcomes regarding the prognostic role of endometriosis in OCCC [40, 59–63]. In our study, OCCC with endometriosis origin showed a trend toward improved survival outcomes. OCCC with endometriosis was found younger, more in early stage, more referred after incomplete surgery due to its unexpectedly diagnosis during surgery for young women with presumed endometrioma, more presented with intraoperative tumor rupture while had a lower incidence of positive ascites cytology, which is in line with previous studies [30, 60]. There may be a difference in the pathogenesis and underlying biology of OCCC in patients with endometriosis origin. Therefore, further studies are required to explore the molecular mechanisms of pathogenesis, molecular genetic features of OCCC derived from endometriosis.

## Conclusions

In conclusion, patients with ovarian clear cell carcinoma are younger, tend to present at an early stage, tumors with or without endometriosis origin have different clinical features in many aspects. Genetic, epigenetic, metabolic and immunological factors interact or combine with each other and are induced or directly influenced by specific microenvironments to lead to the development of OCCC. The early stage and proper Chinese herbal medicine treatment postoperatively are important independent factors to improve patients’ prognosis. While the non-necessity of lymphadenectomy in advanced ovarian cancer has been proven, we here again question the necessity of lymphadenectomy in the early stage ovarian cancer. A multi-center clinical trial is currently underway in China and its results will be used to guide gynecologic surgeon in deciding the scope of surgery and selecting proper regimen for their patients. Surely, our study in the present has several limitations, which include the potential inherited unmeasured biases associated with its retrospective nature, the small sample size, single-institution study and variable follow-up length. Hence, larger-scale, prospective, randomized and well-controlled studies are required to confirmed the findings presented herein.

## Data Availability

The original contributions presented in the study are included in the article material. Further inquiries can be directed to the corresponding author.

## Abbrevations

OCCC: Ovarian clear cell carcinoma
FIGO: The International Federation of Obstetrics and Gynecology
OS: Overall survival
PFS: Progression-free survival
EOCs: Epithelial ovarian cancers
HGSOC: High-grade serous ovarian cancer
WHO: World Health Organization
CNV: Copy number variation
PD-L1: Programmed cell death-ligand 1
BMI: Body mass index
MRI: Magnetic resonance imaging
PET-CT: Positron emission tomography-computed tomography
CA-125: Carbohydrate antigen 125
SPSS: Statistical Program for Social Sciences
HR: Hazard ratio
CI: Confidence interval
OB/GYN: Obstetrics and gynecology
PARP: Poly(ADP-ribose) polymerase
